# Development of a Risk Score to Aid With the Diagnosis of Infections After Spinal Cord Injury: Protocol for a Retrospective Cohort Study

**DOI:** 10.2196/52610

**Published:** 2025-05-08

**Authors:** Felicia Skelton, Larissa Grigoryan, Joann Pan, Ashley Collazo, Barbara Trautner

**Affiliations:** 1 Center for Innovations in Quality, Effectiveness and Safety (IQuESt) Michael E. DeBakey VA Medical Center Houston, TX United States; 2 H. Ben Taub Department of Physical Medicine and Rehabilitation Baylor College of Medicine Houston, TX United States; 3 Department of Family and Community Medicine Baylor College of Medicine Houston, TX United States; 4 School of Medicine Baylor College of Medicine Houston, TX United States; 5 Section of Health Services Research Department of Medicine Baylor College of Medicine Houston, TX United States

**Keywords:** spinal cord injury, neurogenic bladder, asymptomatic bacteriuria, positive predictive value, risk assessment, diagnosis, SCI, risk score, urinary tract infection, UTI, quality of life, complication, urosepsis, management, vital sign, retrospective, cohort, physiology, bladder health, bladder, urinary tract, infection

## Abstract

**Background:**

Patients with spinal cord injury (SCI) who develop urinary tract infection (UTI) present differently than the non-SCI population. UTIs can cause loss of quality of life and even lead to life-threatening complications including urosepsis. Challenges in SCI management include distinguishing symptomatic UTI from asymptomatic bacteriuria (ASB), which occurs often in patients with SCI, and the lack of standardization in UTI diagnosis in SCI.

**Objective:**

This study aims to set the foundation for the development of a risk score to improve diagnostic accuracy of UTI after SCI.

**Methods:**

This study will use data from the Veterans Health Administration Corporate Data Warehouse from national outpatient clinics. It will use 2 approaches: (1) a case-control study comparing frequency-matched healthy SCI cases (n=2000) with healthy non-SCI controls (n=2000) to establish a physiologic baseline for vital sign and lab measurements after SCI and (2) a retrospective cohort study of patients with SCI (n=400) to determine the positive predictive value of the baseline vital signs and lab measurements found in step 1, from which a threshold for clinically meaningful UTI after SCI will be established.

**Results:**

The study was funded in May 2023, and initial data extraction started in early 2024 and is expected to be completed in 2026. Data extraction, analysis, and results for aim 1 were completed as of manuscript submission. For aim 1, we hypothesize that SCI will be associated with lower temperature, heart rate, and systolic blood pressure when compared with non-SCI controls. SCI will also be associated with higher baseline levels of pyuria and peripheral white blood cells when compared with non-SCI controls. Data extraction for aim 2 will begin in year 1, and analysis and results will be completed in year 2. For aim 2, we hypothesize that pyuria, heart rate, and temperature measurements will have a high positive predictive value for clinically meaningful UTI.

**Conclusions:**

Once complete, this study will be the basis for our future work developing a risk score to aid with the diagnosis of UTI after SCI and prevent antibiotic overuse in patients with SCI.

**International Registered Report Identifier (IRRID):**

DERR1-10.2196/52610

## Introduction

### Urinary Tract Infection After Spinal Cord Injury

After spinal cord injury (SCI), urinary tract infection (UTI) poses significant economic burden and interruption to quality of life [1]. UTI, including catheter-associated UTI (CAUTI), is a common and costly medical complication after SCI [1]. Our research team and others have found that diseases of the genitourinary tract, of which a large proportion are UTI or CAUTI, are among the most common reasons for emergency room and inpatient visits, with septicemia being one of the leading causes of death for persons with SCI [1,2]. UTI and CAUTI are the focus of multiple quality improvement campaigns, with many hospitals and health care facilities setting their sights on “Getting to Zero” [3]. Some of the impetus for preventing CAUTIs has been attributed to the change in the Centers for Medicare and Medicaid reimbursement policy in 2008 [4], which provided hospitals financial incentive to prevent CAUTIs as an important component of hospital quality metrics [5].

Contrary to common belief, walking after SCI is not among the most important indicators of quality of life, but adequate control of bladder function (including minimizing the disruption of UTI or CAUTI) is [6-8]. Therefore, providers, patients, and hospital systems all have a vested interest in accurately identifying this burdensome condition. For simplification, UTI will be used throughout this paper to represent UTI and CAUTI in this population.

### UTI Versus Asymptomatic Bacteria

Distinguishing UTI from asymptomatic bacteria (ASB) after SCI is challenging, often leading to antibiotic overuse and its harms. The rate of ASB, also defined as bacteriuria in the absence of signs or symptoms of infection, in persons with SCI is very high due to how common bladder instrumentation is in this patient population and does not require antibiotic treatment [9]. For many clinicians, however, translating ASB and UTI treatment guidelines to the bedside proves difficult when faced with a “positive” urinalysis or urine culture result [10,11]. In a pilot study, our research team demonstrated that a positive urine culture led to 35% of ASB cases being treated with unnecessary antibiotics in the SCI population [12]. Overtreatment of ASB promotes the development of multidrug-resistant organisms and *Clostridioides difficile* infections, both of which occur frequently in the SCI population [13-16].

Accurately distinguishing between UTI and ASB based on clinical practice guidelines is cognitively taxing in the non-SCI population; the challenge is even greater for accurately diagnosing UTI in persons with SCI [17]. Persons with SCI often do not present with typical infection signs and symptoms (ie, dysuria, suprapubic pain, urinary frequency) due to lack of sensation and bladder management strategy. The Infectious Diseases Society of America (IDSA) guidelines include SCI-specific signs and symptoms such as increased spasticity and autonomic dysreflexia [18] in their decision algorithms for UTI diagnosis [19], but limited evidence on the sensitivity and specificity of these symptoms exists.

Patients and providers alike have difficulty determining which signs and symptoms arise from UTI. Several studies have shown that patients with SCI are often inaccurate when identifying when an UTI is present, with an accuracy rate between 61% and 66% [20,21]. Okamoto et al [22] performed a qualitative study of intermittent catheterization users to understand their views of signs and symptoms of UTI, finding that users had difficulty distinguishing UTI symptoms from symptoms of their comorbidities. Our research team recently completed a mixed methods analysis exploring Veterans Health Administration (VHA) SCI providers’ knowledge and attitudes toward distinguishing ASB from UTI with nearly one-third of all participants endorsing non-IDSA guideline–recommended triggers for obtaining a urine culture (ie, change in urine color, cloudiness, or odor) [23]. The type of organism identified on culture drove unnecessary antibiotic use; 57% would treat ASB if caused by extended-spectrum beta-lactamase *Escherichia coli* [23]*.* Interview analysis identified how the limited recall of clinical practice guidelines for ASB and UTI content among SCI providers was a potential barrier to their use [23].

An antibiotic stewardship program is defined as “a multidisciplinary activity that includes appropriate selection, dosing, route, and duration of antimicrobial therapy” [24]. The Centers for Disease Control and Prevention has called for immediate, nationwide improvements in infection control and antibiotic prescribing to prevent increasing drug-resistant infections [25]. The United Nations recently convened a general assembly to address the global concern of antibiotic resistance, the fourth instance of such a high-level meeting for a health issue since its inception [26]. Therefore, a risk score that improves the accuracy of UTI diagnosis and minimizes the overtreatment of ASB would be a high-value stewardship tool in the SCI population.

### Impact of Cardiovascular and Immunologic Changes

Quantification of the impact of cardiovascular and immunologic changes on vital signs and routine laboratory tests is a knowledge gap. The pathophysiology of autonomic nervous system changes leading to cardiovascular abnormalities after SCI have been well described [27,28], but there is little information quantifying the degree of change or expected values for vital sign readings in the SCI population. Disruption of the sympathetic nervous system in cervical and high thoracic level injuries leads to vasodilation and orthostatic hypotension, risk of autonomic dysreflexia, bradycardia, and impaired thermoregulation [28]. In order to develop an accurate diagnostic tool to identify severe UTI, having a better sense of the expected values of vital sign readings for the various neurologic levels of SCI is crucial.

Numerous authors have described the multi-organ dysfunction and systemic inflammation present after SCI. The genitourinary tract is especially affected due to the development of neurogenic bladder, kidney damage, and UTI [29,30]. SCI-induced immune depression is thought to be caused, in part, by disruption of the autonomic nervous system [31]. There is emerging evidence that episodes of autonomic dysreflexia also contribute to immune suppression after SCI [32]. In summary, what is normal for a person with SCI, in terms of vital signs and routine lab results, may differ at baseline from similar signs and laboratory measurements in the non-SCI population. Determining whether derangements in vital signs and lab tests in a person with SCI are indicative of UTI requires that we first establish what is normal in persons with SCI.

### Identifying Baseline Physiologic Measurements

The impact of SCI on baseline vital sign and laboratory values has not been determined or measured systematically once the patient is beyond the acute injury. Most of the baseline physiologic measurements used to diagnosis abnormality in SCI are extrapolations from the non-SCI population [32,33]. Quantifying the normal values in chronic SCI will help investigators answer further questions about the autonomic and immunologic influences of SCI and its impact on diagnosing UTI after SCI.

### Framework for Identifying Clinically Meaningful Infections

This study draws on established concepts of identifying and stratifying disease severity and infection requiring hospitalization. The Acute Physiology and Chronic Health Evaluation-II (APACHE-II) score uses a point score based on the initial values of 12 routine physiologic measurements, age, and previous health status to provide a general measure of the severity of disease. The score can also be tracked over time to predict mortality. Vital sign values such as temperature, heart rate, and respiratory rate and laboratory values such as white blood count and serum creatinine are given points depending on whether they are in the high abnormal or low abnormal range [34]. As described, however, physiologic ranges for these measurements after SCI have not been established.

To bridge this gap in the literature, this study aims to (1) establish physiological baseline vital sign measurements after SCI and (2) determine the positive predictive value (PPV) of those baseline vital signs and lab measurements and to create a threshold for clinically meaningful UTI after SCI.

## Methods

### Rationale

**Figure 1:**
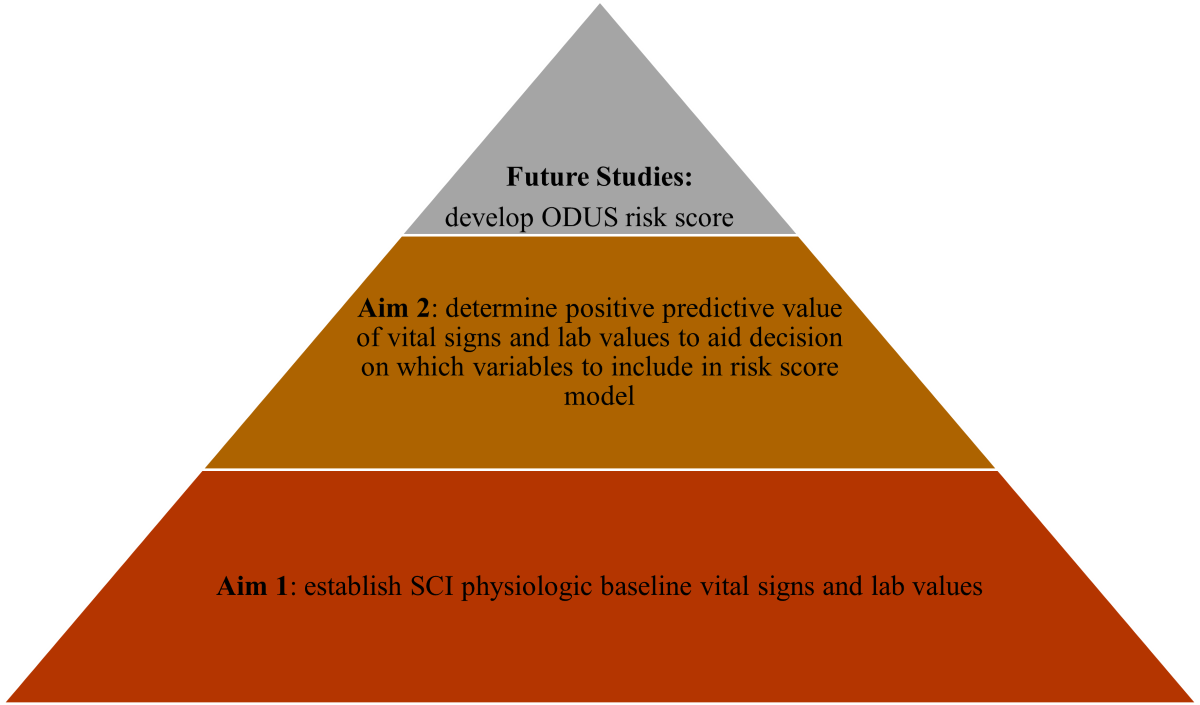
Building the foundation toward the objective diagnosis of urinary tract infection after spinal cord injury (SCI; ODUS) risk score.

Figure 1 provides the rationale for each aim proposed in this study, as well as how the work of this study will guide further research. The aims build upon, but are not dependent on, each other and will support the robust development of a risk score to help make the diagnosis of UTI after SCI more objective.

### Aim 1 Data Extraction (Year 1)

Data will be extracted from the national Veterans Health Administration (VHA) Spinal Cord Injuries & Disorders (SCI/D) Registry (SCIDR) and VHA Corporate Data Warehouse (CDW) from 2018 through 2019 to establish the physiologic baseline for vital sign and lab measurements after SCI. SCIDR is a large SCI/D registry used as a case identification platform for VHA patients that can directly be linked to CDW and all other VHA health care data [35]. CDW is a national repository that includes clinical and administrative data from VHA. Data are stored in a relational database (ie, data are grouped into several domains that are structured to recognize relations among the stored information) and are updated on a continual basis [36]. We will use data from these domains within CDW to obtain the vital signs and laboratory values listed in Table 1. Urinalysis tests are done routinely with healthy veterans with SCI due to national guidelines [37] and not infrequently with other veterans within Veterans Affairs (VA) due to the high incidence of genitourinary concerns [38]. Sociodemographic data including age, race, ethnicity, neurologic level of injury, sex, and medical comorbidities will be used to construct a Deyo comorbidity index.

**Table 1:** Vital sign and laboratory values to be examined.

Outcome variable name			Type of variable		Comments
**Vital signs**					
	Systolic blood pressure	Continuous		This is found in the CDW^a^ Outpatient Vital Sign Domain.	
	Diastolic blood pressure	Continuous		This is found in the CDW Outpatient Vital Sign Domain.	
	Heart rate	Continuous		This is found in the CDW Outpatient Vital Sign Domain.	
	Temperature	Continuous		This is found in the CDW Outpatient Vital Sign Domain.	
	Respiratory rate	Continuous		This is found in the CDW Outpatient Vital Sign Domain.	
**Urinalysis results**					
	White blood cell count	Continuous		This is found in the CDW Micro Domain.	
	Red blood cell count	Continuous		This is found in the CDW Micro Domain.	
	pH	Continuous		This is found in the CDW Micro Domain.	
	Nitrites	Categorical (present/absent)		This is found in the CDW Micro Domain.	
	Leukocyte esterase	Categorical (present/absent)		This is found in the CDW Micro Domain.	
**Peripheral blood results**					
	White blood cell count	Continuous		This is found in the CDW Outpatient Lab Domain.	
	Hemoglobin	Continuous		This is found in the CDW Outpatient Lab Domain.	
	Platelets	Continuous		This is found in the CDW Outpatient Lab Domain.	
	Creatinine	Continuous		This is found in the CDW Outpatient Lab Domain.	
	Blood urea nitrogen	Continuous		This is found in the CDW Outpatient Lab Domain.	
	Glucose	Continuous		This is found in the CDW Outpatient Lab Domain.	

^a^CDW: Corporate Data Warehouse.

### Aim 1 Approach

We used a case-control retrospective study design. We included healthy Veterans with SCI (cases: n=2000) as well as healthy Veterans without SCI (controls: n=2000), seen in the outpatient clinic setting in 2018 and 2019. The cases and controls were randomly sampled and frequency matched based on the variables listed in Table 2. Cases and controls were excluded if the encounter has any International Classification of Diseases, Tenth Revision (ICD-10) code for an infectious disease (see Multimedia Appendix 1). Cases were adults aged 18 years and older with an SCI diagnosis confirmed via the SCIDR registry plus the presence of an SCI/D Annual Exam. Controls were adults without an SCI diagnosis confirmed by ensuring they were not on the SCID Annual Exam Reports. This study will conform to all STROBE (Strengthening the Reporting of Observational Studies in Epidemiology) guidelines for a case-control study and will report the required information accordingly. 

**Table 2:** Variables for frequency matching of cases and controls.

Variable name	Type of variable	Comments
Age	Continuous	This is found in the CDW^a^ Age domain.
Sex	Categorical (male/female)	This is found in the CDW Sex domain, and we oversampled female SCI^b^ cases to reflect the general population.
Race	Categorical (white/Black/other)	This is the convention we have used in previous studies based on the typical distribution of the study population.
Ethnicity	Categorical (Hispanic/not Hispanic)	This is found with the domains.
Deyo comorbidity index	Continuous	This is generated by using medical comorbidities extracted from CDW domains.

^a^CDW: Corporate Data Warehouse.

^b^SCI: spinal cord injury.

### Aim 1 Statistical Analysis

The primary exposure is SCI and categorical. Descriptive statistics for each group will be calculated, including mean and SD (continuous variables) and frequency counts and percentages (categorical variables). Depending on the distribution, *t* tests or Mann-Whitney *U* tests will be used to compare the means of continuous variables of the cases to those of the controls. Chi-square tests will be used to compare categorical variables between cases and controls. Each laboratory variable will be regressed on a dummy variable indicating group membership (SCI vs non-SCI) along with potential confounding variables including age, gender, race/ethnicity, and neurologic level of injury for the cases (ie, tetraplegia and paraplegia), and standard model assessment practices (eg, examination of residuals) will be used to ensure assumptions are met and the logistic regression model is appropriate [39]. Each regression model will serve 2 purposes: (1) A test of the group membership indicator will assess group differences between patients with SCI and control patients, and (2) using marginal effects analysis, population marginal means and their confidence intervals will be derived from the models to serve as covariate-adjusted estimates for normed values among patients with SCI [40]. Marginal effects make logistic regression analysis results more easily understood, by expressing the change in probability that the outcome occurs as the risk factor changes by 1 unit while holding all the other explanatory variables constant [40].

There are numerous methods to determine a minimal clinically important difference (MCID), and each method has its pros and cons [41]. We will use the distribution-based method for our study, because it considers measurement error, is readily implemented, and is easy for others to understand. There is no golden criterion to judge MCID by distribution method so far. Based on work by Kallogjeri et al [42], we will use these definitions for MCID: 0.2 SD as a small difference, effects around 0.5 SD as moderate, and effects as large as 0.8 SD or larger as large differences.

With the anticipated sample size drawn from CDW, we expect 80% with an effect size of 2% after accounting for variance explained by as many as 10 confounding variables and an α of .05. This is in accordance with the commonly cited guideline for a small effect size [43].

### Aim 2 Data Extraction (Year 1-2)

Data for Aim 2 will also be extracted from CDW outpatient encounters from 2018 to 2019 to determine the PPV of the physiologic vital sign and lab measurements for clinically meaningful UTI after SCI. SCI diagnosis will again be confirmed using the SCIDR Registry. The same variables will be extracted from CDW domains as listed in Table 1. In addition, urine culture results, signs and symptoms of UTI, and bladder management strategies (ie, spontaneous voiding, indwelling catheters-intraurethral, intermittent catheterization) will be extracted from CDW through in-depth chart review by 2 members of our study team. Basic demographic and comorbid conditions will also be extracted from CDW as described in Aim 1.

### Aim 2 Approach

We will use a retrospective cohort study design. The cohort will include a national random sample of Veterans with SCI seen in the outpatient setting (n=400). Through chart review of the electronic health record, each eligible encounter will be classified as having ASB, having a UTI, having neither, or indeterminate using a validated algorithm developed by our team and previously used in this patient population; it showed high reliability between expert and nonexpert clinicians when the algorithm was used [17,44]. The algorithm incorporates IDSA guidelines for UTI symptomatology in SCI as well as the IDSA recommended interpretation of culture results based on the organisms per mL reported on the urine culture [44]. The first step of the validated algorithm is to determine the presence of signs or symptoms of UTI, which our team will determine through examination of the electronic medical record. We will continue to follow the algorithm by referring to encounters during the study time period until a final classification is assigned for each person in the cohort. This study will conform to all STROBE guidelines for a cohort study and will report the required information accordingly.

### Aim 2 Statistical Analysis

Using the moderate MCID of vital sign and laboratory values determined from Aim 1, the PPV will be calculated as the ratio of the proportion of all those screened who meet the criteria for UTI and a positive test (ie, moderate MCID value for blood pressure and heart rate) to the proportion of all those screened who have a positive test. A similar analysis method was used by Massa et al [20] to determine predicative signs and symptoms of UTI after SCI. The 95% CIs of the PPV will be calculated using a binomial distribution.

The sample size was calculated using a 30% expected prevalence of UTI and a 0.05 precision.

### Ethical Considerations

This study has institutional review board approval, which covers secondary analysis without additional consent from Baylor College of Medicine (H-50481) and the Veterans Health Administration (22A07.H). An informed consent waiver was approved by the institutional review board, as data will be deidentified upon extraction to protect patient privacy.

## Results

### Study Progress

This study was funded in May 2023, with initial data extraction starting in early 2024 and expected completion in 2026. Data extraction, analysis, and results for the 4000 records (cases: n=2000; controls: n=2000) for Aim 1 were completed upon submission of this manuscript. Data extraction for Aim 2 began in year 1, and analysis and results will continue in year 2.

### Dissemination Plan

Plans for dissemination include presentation of results at SCI and Physical Medicine and Rehabilitation conferences, sharing findings with the Paralyzed Veterans of America and local Veterans Engagement Board, and sharing results with VA SCI team members through cyber seminars.

## Discussion

### Principal Considerations and Anticipated Results

There is a pressing need for more targeted and efficient diagnosis and management of UTIs in the SCI population. SCI-specific tools and protocols can be invaluable for achieving better patient outcomes, reducing unnecessary antibiotic treatments, and improving the overall quality of life for these patients. There is not currently a standardized risk score for identifying UTIs in the SCI patient population, which this study aims to address.

#### Aim 1 Anticipated Results

We anticipate that mean blood pressure, heart rate, temperature, serum creatinine levels, and hemoglobin levels will be lower in the SCI cases than in the non-SCI controls. Mean serum white blood count, glucose levels, and urine white blood cells will be higher in the SCI cases than in the non-SCI controls.

#### Aim 1 Potential Pitfalls and Alternative Approaches

Extracting and refining data from relational databases can be challenging, but our entire study team has experience with doing so. The principal investigator, who is also the data analyst, for this proposal has been extracting SCI-specific data for her current career development award; over 7000 SCI cases have been identified with the desired values. Because of the large number of SCI cases, we will be able to oversample for women and minorities to overcome the limitations of the VA SCI population (largely male and white) to make our sample more representative of the general population. The CDW database results will be validated by chart review of 100 cases and 100 controls for accuracy.

Even if no difference is found in vital sign and lab measurements for people with SCI compared with non-SCI controls, this will be a valuable contribution to the field of knowledge, and Aim 2 can still proceed with a solid foundation on this knowledge.

#### Aim 2 Anticipated Results

The PPVs of moderately MCID pyuria, systolic blood pressure, and temperature will be high for UTI after SCI.

#### Aim 2 Potential Pitfalls and Alternative Approaches

By using a nationwide sample, we will have an abundance of SCI cases, allowing us to oversample for women and minorities to overcome the limitations of the VA SCI population (largely male and white) in order to make our sample more representative of the general population. By taking this rigorous approach, we will ensure that the variables with the highest PPV will be included in the development of the risk score*.*

### Comparison With Previous Work

Although previous work looked at and showed fever and autonomic dysreflexia having high sensitivity (99%) for UTI in the SCI patient population [20], this study will look more closely at specific lab values and vitals, such as urine and serum white blood cells in a much larger patient population. This study aims to first establish baseline labs including various urine and blood serum markers and vitals after SCI injuries, such as temperature and heart rate. The study then will create an objective risk score to better help differentiate between ASB and UTI in the SCI patient population.

### Current Limitations

The VA SCI population will not be fully representative of the entire patient population with SCI injuries. There will also be limitations inherent to use of the electronic health record data [45]. The accuracy and reliability of the encounter data are reliant on the diligence of the clinician or health care professional imputing the information. However, any human error in data input would likely be random and not systematic in nature, resulting in only a dilution of our study findings. As further limitations arise in our study, we aim to address them.

### Conclusion

This study will provide the foundation for our future work of developing a risk score to help improve accuracy of the diagnosis of clinically meaningful UTI after SCI. This work in turn will help guide antibiotic use in the SCI patient population, providing much needed stewardship in this vulnerable population and preventing antibiotic overuse in this population.

## Appendix

Multimedia Appendix 1 Exclusion ICD-10 Codes for Infectious Diseases.

## Abbreviations

APACHE-II: Acute Physiology and Chronic Health Evaluation-II
ASB: asymptomatic bacteria
CAUTI: catheter-associated urinary tract infection
CDW: corporate data warehouse
ICD-10: International Classification of Diseases, Tenth Revision
IDSA: Infectious Diseases Society of America
MCID: minimal clinically important difference
PPV: positive predictive value
SCI: spinal cord injury
SCI/D: Spinal Cord Injuries & Disorders
SCIDR: Spinal Cord Injuries & Disorders Registry
STROBE: Strengthening the Reporting of Observational Studies in Epidemiology
UTI: urinary tract infection
VHA: Veterans Health Administration

## Data Availability

Data sharing is not applicable to this article as no data sets were generated nor analyzed during this study.
