# Supplementary material for: Development of a Risk Score to Aid With the Diagnosis of Infections After Spinal Cord Injury: Protocol for a Retrospective Cohort Study
Source: JMIR Res Protoc. 2025 May 8;14:e52610. doi: 10.2196/52610 (PMC12099271; doi:10.2196/52610)
Supplement: Multimedia Appendix 1 [file resprot_v14i1e52610_app1.docx]

| Exclusion ICD-10 Codes for Infectious Disease [1] | |
| --- | --- |
| Diagnoses | ICD10 |
| Upper Respiratory Infections | J00, J01.90, J02.9, J03.90, J06.9, J31.0, J32.9, R05 |
| Lower Respiratory Infections | A37.90, B97.4, J18.9, J20.9, R06.02, R09.1, R76.11 |
| GI Infections | A03.9, A04.7, A05.9, A07.1, B89, B96.20, B96.29, B96.81, K21.0, K29.00, K29.70, K29.9, K30, K31.9, K50.90, K52.9, K57.30, K58.0, K58.9, K59.00, K85.9, K92.1, K92.2, R10.11, R10.12, R10.13, R10.31, R10.32, R10.84, R10.9, R14.0, R14.1, R14.2, R14.3, R19.7, R19.8 |
| Liver | B17.10, B18.1, B18.2, B19.20, F15.10, F18.10, F19.10, K72.10, K72.11, K74.60, K75.9, K76.0, K76.9 |
| Skin/Wound Infections | B02.9, B35.1, B95.62, L02.419, L02.91, L03.119, L03.90, L08.89, L08.9, L20.89, R21, T07 |
| Women’s Health | B37.3, N39.0, N72, N76.0, N76.89, N94.9, N95.9, N97.9, O09.893, O09.93 |
| Urinary System and Men’s Health | N30.00, N30.01, N30.90, N30.91, N36.9, N39.0, N39.9, N41.9, N42.9, R30.0, R31.0, R31.2, R32, R35.0, R35.8, R39.15 |
| Vector-Borne Illnesses | A69.20, A77.0, A78.0, A90.0, A91, A92.0, A92.30, B54.0, G05.3 |
| Tuberculosis | A15.0, A15.7, A15.8, A17.0, A18.01, A18.02, A18.03, A18.09, A18.4, A19.9, B90.9 |
| Venereal Diseases | A51.0, A53.9, A54.00, A60.9, A63.8, A74.9, B00.9, B20, B97.7, Z11.3, Z20.2, Z72.51, Z72.52, Z72.53, Z77.21 |
| Other | A41.9, B34.9, B89, B99.9, D70.9, D72.819, D72.829, G03.9, R07.89, R07.9, R11.0, R11.10. R11.2, R50.9, R51, R53.81, R53.82, R53.83, R63.4, T80.29XA, T88.0XXA, Z01.818, Z02.89, Z11.59. Z11.9, Z13.89 |

1. Quest Diagnostics. ICD-10-CM Codes for Infectious Disease. September 2015. Accessed December 16, 2024. [https://www.questdiagnostics.com](https://www.questdiagnostics.com/)
